# Blind testing of cross‐linking/mass spectrometry hybrid methods in CASP11

**DOI:** 10.1002/prot.25028

**Published:** 2016-03-28

**Authors:** Michael Schneider, Adam Belsom, Juri Rappsilber, Oliver Brock

**Affiliations:** ^1^ Robotics and Biology LaboratoryTechnische Universität Berlin10587BerlinGermany; ^2^ Wellcome Trust Centre for Cell BiologyUniversity of EdinburghEdinburghEH9 3BFUnited Kingdom; ^3^ Department of BioanalyticsInstitute of Biotechnology, Technische Universität Berlin13355BerlinGermany

**Keywords:** blind test, hybrid methods, protein structure prediction, CASP, cross‐linking, mass spectrometry

## Abstract

Hybrid approaches combine computational methods with experimental data. The information contained in the experimental data can be leveraged to probe the structure of proteins otherwise elusive to computational methods. Compared with computational methods, the structures produced by hybrid methods exhibit some degree of experimental validation. In spite of these advantages, most hybrid methods have not yet been validated in blind tests, hampering their development. Here, we describe the first blind test of a specific cross‐link based hybrid method in CASP. This blind test was coordinated by the CASP organizers and utilized a novel, high‐density cross‐linking/mass‐spectrometry (CLMS) approach that is able to collect high‐density CLMS data in a matter of days. This experimental protocol was developed in the Rappsilber laboratory. This approach exploits the chemistry of a highly reactive, photoactivatable cross‐linker to produce an order of magnitude more cross‐links than homobifunctional cross‐linkers. The Rappsilber laboratory generated experimental CLMS data based on this protocol, submitted the data to the CASP organizers which then released this data to the CASP11 prediction groups in a separate, CLMS assisted modeling experiment. We did not observe a clear improvement of assisted models, presumably because the properties of the CLMS data—uncertainty in cross‐link identification and residue‐residue assignment, and uneven distribution over the protein—were largely unknown to the prediction groups and their approaches were not yet tailored to this kind of data. We also suggest modifications to the CLMS‐CASP experiment and discuss the importance of rigorous blind testing in the development of hybrid methods. Proteins 2016; 84(Suppl 1):152–163. © 2016 The Authors Proteins: Structure, Function, and Bioinformatics Published by Wiley Periodicals, Inc.

**Abbreviations**
CASP: Critical Assessment of Protein Structure Prediction
sulfo‐SDA: sulfosuccinimidyl 4,4′‐azipentanoate
CLMS: cross‐linking/mass spectrometry
FDR: false discovery rate
SAXS: small angle x‐ray scattering
BS3: Bis(sulfosuccinimidyl)suberate.

## INTRODUCTION

Hybrid methods are emerging as new tools to model protein structure. These methods incorporate experimental data into computational protein structure approaches in an attempt to increase the accuracy of resulting models and the range of applicability. Hybrid methods can leverage experimental data that by itself would be insufficient to determine structures with satisfactory accuracy. However, when this data is complemented by computational approaches, it may suffice to aid conformational search to find good minima in the energy landscape, even in cases when purely computational methods would fail.

Experimental data sources for hybrid methods range from sparse NMR restraints,^1^ low‐resolution electron density data,^2^, ^3^ restraints from electron paramagnetic resonance,^4^, ^5^ Förster resonance energy transfer,^6^ small angle X‐ray scattering data (SAXS),^7^ and cross‐link/mass‐spectrometry data.^8^, ^9^, ^10^, ^11^, ^12^ The simultaneous use of multiple data sources can further increase the accuracy of the resulting model structure.^13^ For a comprehensive review of the protein systems that have been determined with hybrid methods, please refer to Sali *et al*.^14^ In addition, hybrid methods provide models that are experimentally verified and therefore arguably more trustful models of protein structure. Most importantly, many protein targets are elusive to X‐ray crystallography or NMR spectroscopy, because they cannot be isolated with the required purity, are insoluble, or do not crystallize.^14^ However, many experimental methods are still able to collect valuable, structural data on these targets. Thus, hybrid methods are a promising approach for determining structures that are out of reach for established structure determination techniques and expanding our knowledge about the protein universe.

The importance of hybrid methods was acknowledged by the CASP committee in CASP10, when they introduced the “contact‐assisted” category.^15^ In this category, the CASP committee provided sparse contact data (selected from known native contact maps) for difficult modeling targets to mimic distance restraints from hybrid methods. In many cases, this additional information substantially improved the accuracy of protein models over unassisted predictions.^16^ However, the provided contact sets had idealized properties. The sparse contact sets contained long‐range contacts (in terms of sequence separation) that were missed by unassisted predictions and evenly distributed over the protein.^15^ This does not capture the properties of real, experimental data that might be sparse, noisy, ambiguous, and unevenly distributed over the protein. Therefore, algorithms that succeed with the contact sets from the CASP10 contact‐assisted experiment, which might be a best‐case scenario, might not be effective with real experimental data. Obviously, the best benchmark test of hybrid methods is to use real experimental data. However, the CASP experiment imposes time constraints that make it difficult to use real experimental data. Typically, only few weeks to months are available from target selection to the prediction deadline. Most experimental methods need more time to gather sufficient experimental data.

For CASP11, the Brock and Rappsilber laboratory proposed a new experiment to establish hybrid methods as a component of the CASP experiment. To address the time constraints of CASP, they proposed to use experimental data based on a novel protocol for photo‐cross‐linking and mass‐spectrometric analysis (CLMS).^17^ This protocol, as will be described below, promised to deliver valuable structural information obtained from experiments within the required timeframe. Even though the two labs proposed this experiment together, they acted as separate entities in CASP11. The Brock laboratory participated as a prediction group and the Rappsilber laboratory provided the CLMS data to the CASP consortium. During CASP 11, the Brock laboratory only had access to the data released by the CASP consortium. The CASP experiment remained blind in the sense that the Rappsilber laboratory did not know the structure of the proteins for which it was determining experimental cross‐linking data.

The employed photo cross‐linking/mass spectrometry approach in this experiment has a number of unique properties that makes it an excellent experimental data source for blind testing of hybrid methods. Cross‐linking and mass‐spectrometric analysis are relatively quick. The experiments reported in this article took approximately two weeks of experimental time and 4.2 days measurement time on average. This makes it possible to provide experimental data under the time constraints of the CASP experiment. The unique chemistry of photo‐cross‐linking reagents produce an order of magnitude more cross‐links than standard homobifunctional cross‐linking agents, such as BS3.^17^ In favorable cases, this approach can measure 2.5 cross‐links per residue, which approaches the constraint density of NMR (∼3–20 constraints per residue). However, the spatial resolution of the cross‐link constraints is much lower than NMR constraints. Thus, the experimental data from high‐density cross‐linking/mass spectrometry experiments needs to be complemented with structure prediction algorithms to determine protein structure.

Another **important property** is that the cross‐linking reaction can be performed prior to purification of the protein. Because cross‐links are already formed, the protein can be purified under non‐native conditions or the protein can be digested and the cross‐linked proteins can be enriched. Therefore, cross‐linking can be done in samples with low purity, in native environments,^17^ and even in cells.^18^ This enables the gathering of experimental data under conditions that are unsuitable for other experimental methods.

In this article, we describe the first blind test of a hybrid method in CASP with real, experimental data.

We would like to point out that this was made possible by the efforts of the CASP organizing committee. The CASP committee identified and acquired suitable targets and published the resulting data on the CASP web page for the community of predictors. We would also like to thank the experimental groups that generously provided protein sample for this experiment. Please refer to the “Acknowledgements” section a full list of the involved researchers and affiliations.

The goal of this article is to report on the experience and the logistics of the blind testing of hybrid methods. Furthermore, we report on the results of the experiment in CASP11 by presenting the cross‐linking results on four protein targets and briefly discuss the impact on modeling. The last goal of this article is to make recommendations to maximize impact of future instances of hybrid methods in CASP.

## METHODS

Here, we only give a brief overview of the experimental cross‐linking/mass spectrometry method, describing those details required for understanding the remainder of this article. Full experimental details of the experimental protocol used in CASP 11, targeted to a mass spectrometry audience, can be found in a separate article (currently in preparation).

### General overview of high‐density cross‐linking/mass spectrometry

**Figure 1:**
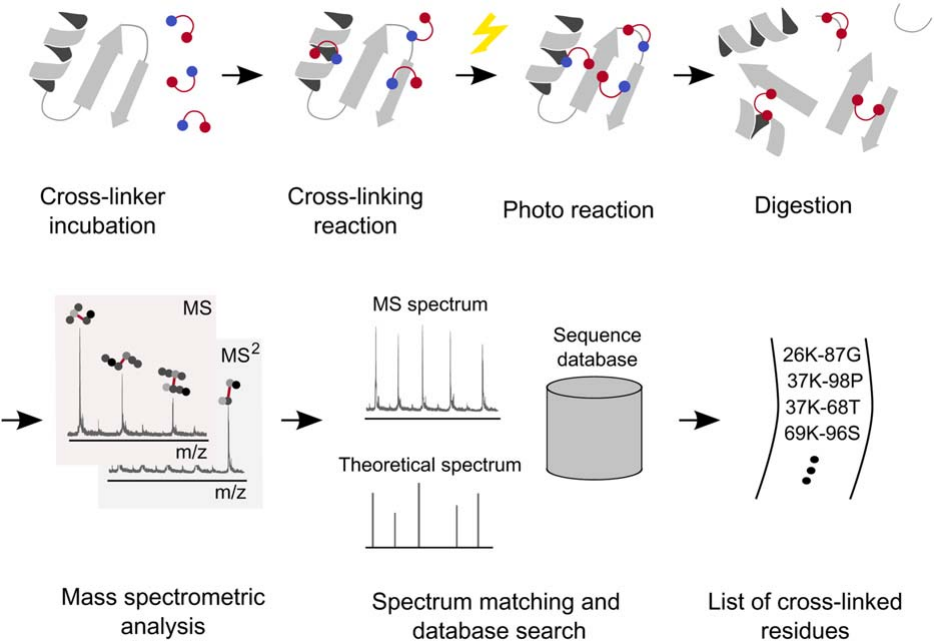
Schematic summary of high‐density cross‐linking/mass spectrometry experiments in CASP11. We incubate the target protein with the sulfo‐SDA cross‐linker. During incubation, the cross‐linker reacts with lysine, serine, threonine, tyrosine, and the protein N‐termini at one end. Upon activation with UV light, the other side of the linker forms a reactive carbene species and reacts with any other amino acid in close proximity. We then digest the protein using proteases. In the analysis step, we subject the peptide mixture to mass spectrometric analysis. We match the mass spectra to theoretical spectra of sequence fragments derived from the target sequence. The output of this procedure is a list of cross‐linked residues.

Generally, protein residue pairs are covalently cross‐linked, effectively providing an upper bound of the linked residues that is partially determined by the length of the linker agent. The protein is then digested, which results into a peptide mix. Some of the peptides are cross‐linked if they have been in spatial proximity in the folded structure. The peptide mix is then subjected to mass spectrometric analysis. Peptide spectrum matching and database search reveals the cross‐linked residues. The output of this method is a list of cross‐linked residues which effectively provide distance restraints with an upper distance bound (Fig. 1, a detailed review of the cross‐linking/mass spectrometry process has been published elsewhere^19^). The key component of our high‐density cross‐linking method is a highly reactive, photoactivatable cross‐linking reagent, sulfo‐SDA. Sulfo‐SDA contains a diazirine group, which releases highly reactive carbene under UV‐light activation that is able to react with any amino acid. This broad specificity greatly increases the number of cross‐links over standard cross‐linking reagents with specific reactivity profiles, effectively resulting in a high number of cross‐links.^17^

### Chemical cross‐linking

Each target was cross‐linked using the heterobifunctional, photoactivatable, chemical cross‐linker sulfosuccinimidyl 4,4′‐azipentanoate (sulfo‐SDA). The Rappsilber group first incubated sulfo‐SDA with the protein for 1 h and then photoactivated the sulfo‐SDA with UV light. The protein is then digested using different combinations of proteases.

### Mass spectrometry and data analysis

The digested peptides were loaded onto a liquid chromatography column to separate the peptides by hydrophobicity. The peptides were gradually eluted and sprayed into the mass spectrometer. This procedure reduces sample complexity during mass spectrometric analysis.

For data analysis, the peak lists were searched against a database from the sequence of the CASP targets. The Rappsilber lab assumed the sulfo‐SDA linker reaction specificity to be lysine, serine, threonine, tyrosine, and protein N‐termini at one end and any amino acid residue at the other end. Lastly, the false discovery rate (FDR) was estimated using a modified target‐decoy search.^20^, ^21^

### CLMS data release

The Rappsilber group compiled lists of residue‐residue cross‐links from FDR analysis and submitted them to the CASP organizing committee, which released them to the prediction groups.

## RESULTS

### Organization and execution of the cross‐linking experiment

First, we report on the organization and execution of the experiment to provide the reader with the setup of the hybrid method/cross‐linking experiment in CASP11. We proposed the CLMS assisted structure prediction experiment mid‐March 2014.

The CASP organizers identified and acquired suitable protein targets (no homologous structures could be identified by sequence similarity). The first positive response came from a PSI centre 12 days later on March 30th 2014. A total of nine proteins were sent to the Rappsilber laboratory between May 29th and June 9th 2014. From these nine proteins, the CASP organizing committee, in discussion with the Rappsilber group, selected four proteins that met the following criteria: 1) The protein is heavier than > 20 kDa, 2) it forms a monomer in solution and, 3) approximately 1 mg protein sample was available. Because of the relatively low spatial resolution (25 Å) of CLMS constraints, CLMS data is likely not informative for small proteins. Thus, the organizers excluded small proteins from consideration. Selected proteins needed to be monomers in solution to allow unambiguous assignment of cross‐linked peptides as intramolecular connections. At least 1 mg of protein sample should be available to have sufficient material for CLMS experiments. The final conclusion was made in a meeting between the CASP Organizing Committee and the Rappsilber lab Edinburgh on June 10th 2014. The selected targets for CLMS experiments were: Target 1, SP17834A‐RUMGNA_02398, Tx781; Target 2, SP16782A_BACCAC_02064, Tx808; Target 3, SP17984B‐SAV1486, Tx767 and Target 4, laminin, Tx812. All targets contain at least one hard template‐based modeling or free modeling domain.

**Figure 2:**
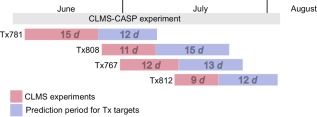
Schedule of CLMS experiments and prediction periods for Tx targets in CASP11. Each colored bar shows the experimental CLMS time (in days, *d*) spent on each target and (red) and the prediction period that this target was available for prediction groups (blue).

The Rappsilber group performed cross‐linking/mass‐spectrometry experiments for these targets in a timeframe of 48 days, starting on June 11 2014. Figure 2 shows the schedule of the CLMS experiments and prediction periods for Tx targets. The Rappsilber group staggered the release of the experimental data to have enough time for data acquisition and be able to work at one target at a time. The expiration dates for the four targets were July 8, July 23, July 28 and August 4 2014, respectively. Prediction groups had between twelve and 15 days to model the proteins between CLMS data release and the expiration of the target. At the time of the experiment, the Rappsilber group had no knowledge of the crystal structure. However, the CASP organizers gave feedback for quality control to rule out complete failure of the CLMS experiments: they released back to the Rappsilber lab the percentage of experimentally determined cross‐links between residues with α‐carbon distance below 20/25 Å in the native structure, i.e. the percentage of plausible cross‐links. CLMS constraints were provided on the CASP website (http://predictioncenter.org/).

### Qualitative analysis of CLMS structure information

Cross‐linking data captures spatial proximity between residue pairs in the native structure. However, the cross‐linked atoms cannot be specified because the carbene species of activated diazirine group can react with any atom and current mass spectrometry technology does not routinely identify the linked atoms. Thus, it is not possible to specify a tight upper bound for the distance between two cross‐linked residues. The actual distance is affected by many factors, such as the side‐chain length, the cross‐linker length, and conformational flexibility of the protein. In CASP11, we used a conservative α‐carbon upper distance bound of 25 Å. Note that conformational flexibility of the protein in solution could result in correct cross‐link matches of residues that are further apart than 25 Å in the native structure.

**Figure 3:**
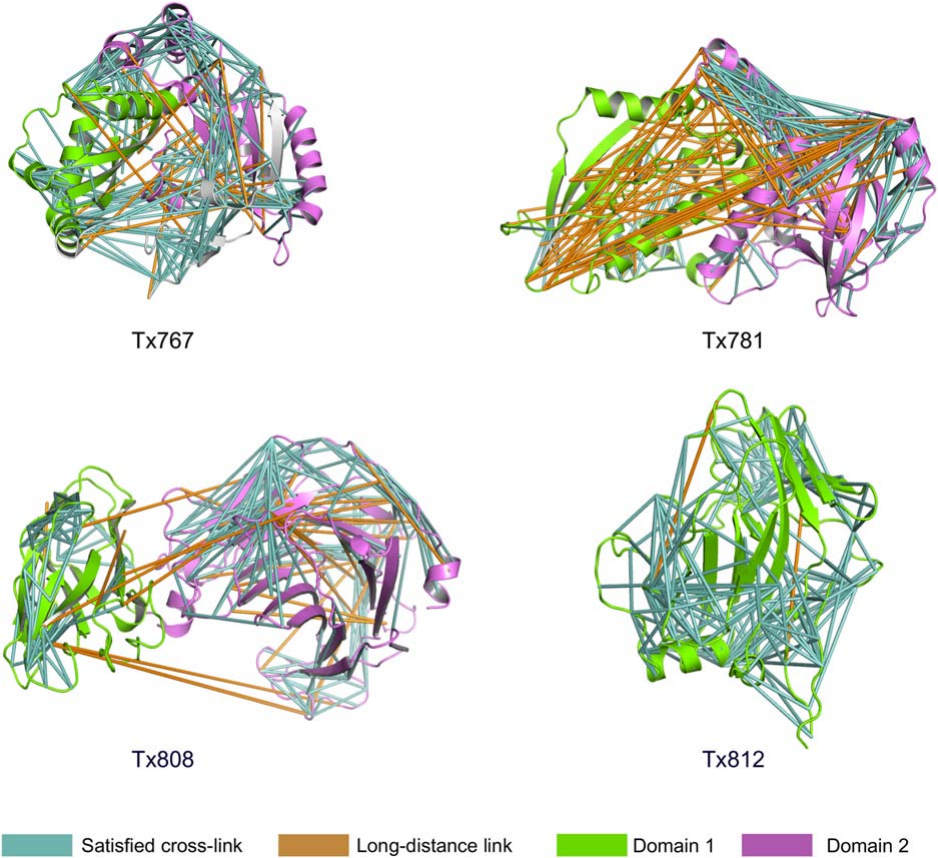
Visualization of cross‐links in target proteins. Cartoon representation of protein target structures with cross‐links obtained by the proposed photo‐CLMS procedure. Cross‐links that satisfy the upper distance bound (<25 Å C_α_‐C_α_ distance) are shown in cyan, long‐distance links that exceed the upper bound in orange. The first domain of the target is shown in green, the second in violet. Tx767: The cross‐links are evenly distributed in the protein. Most long‐distance cross‐links are between domain 1 and domain 2. Tx781: Domain 1 and the domain 1 ‐domain 2 interface contain many long‐distance cross‐links. Domain 2 has more links, but they are unevenly distributed. Tx808: Domain 1 has almost no long‐range cross‐links, but the domain is quite small. Many links can be found in domain 2, but almost no links are identified between β‐strands. Tx812: CLMS experiments produced almost only true‐positive links for this protein. However, there are again almost no links between β‐strands.

We first qualitatively analyze the CLMS data of the four CASP11 target proteins and their evaluation domains. Note that CASP predictions are usually analyzed the basis of evaluation domains that are identified by the assessors. We refer to the official CASP11 domain assignments (see http://www.predictioncenter.org/casp11/domains_summary.cgi) with ‐D1 and ‐D2 for the first and second evaluation domain, respectively. Figure 3 shows the cartoon representation of the crystal structures of Tx767, Tx781, Tx808, and Tx812 with cross‐links indicated as straight‐line connections between residues. The visual inspection of cross‐links provides some interesting insights. Some domains have good coverage of cross‐links (Tx767‐D1, Tx767‐D2, Tx808‐D1, Tx812‐D1). However, Tx808‐D1 and Tx812‐D1 have a sandwich β‐sheet architecture that is slightly elongated. CLMS data will only contain information along the elongation axis, which has a diameter of ∼40 Å for these domains. The diameter perpendicular to the elongation axis is <25 Å for these proteins, which is less than the upper bound for the cross‐linking distance. Thus, CLMS constraints provide no information along this axis.

Another interesting observation is that excessively long‐distance cross‐links are often found between domains. We hypothesize that the flexibility of the domain interface might lead to long‐distance cross‐links, because the CLMS approach captures some domain arrangements in solution that are not seen in the native structure.

We also find that cross‐links are unevenly distributed for some proteins. This is apparent in Tx781‐D2, which contains long stretches without cross‐link coverage. However, we generally find that only few cross‐links are formed between β‐strands. Thus CLMS data might miss the critical information of β‐sheet topology. This is problematic, as the CASP11 targets for the CLMS experiment have significant β‐sheet content (Tx808 and Tx812 are mostly β‐sheets). It is obvious that CLMS constraints between β‐strands are not informative, because adjacent β‐strands have a distance of ∼5 Å. However, it is quite surprising that there is an apparent bias to cross‐link coverage. Specifically, β‐strand cross‐links are often not observed at all. There is no entirely clear reason for this finding at this stage.

Surface accessibility and environmental reactivity influences the formation of cross‐links and maybe a different cross‐linking chemistry needs to be developed to reduce this influence and to obtain a more even coverage.

The distribution of digestion cleavage sites also contributes to uneven coverage of cross‐links. This is evident for Tx781, for which little cross‐link information is found up to residue 180 (see Fig. 3). There are 18 tryptic cleavage sites for the first half of the protein (up to residue 180). In contrast, there are 31 tryptic cleavage sites between residue 181 and the C‐terminus. This reduces the probability of successful digestion of the N‐terminal protein, which could explain the absence of cross‐links. This could be combated with digestion strategies with multiple enzymes that target different cleavage sites.

### Quantitative analysis of CLMS structure information

**Figure 4:**
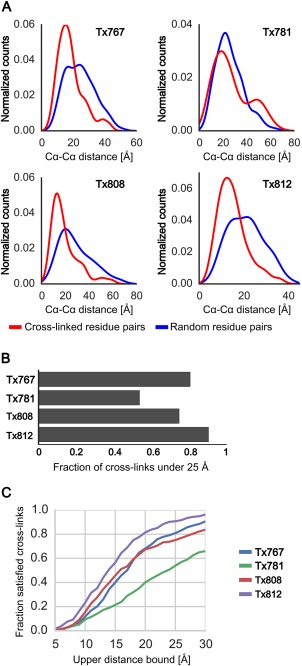
Structural information of CLMS data for four CASP11 targets. Only cross‐links with sequence separation of 12 amino acids or higher are considered for this plot, which results in: 393/332/221/216 links for Tx767/Tx781/Tx808/Tx812. A: α‐carbon distance distribution of cross‐linked residue pairs (red). Distances are taken from the native protein structure. The blue line shows a distance distribution of random residue pairs with the same sequence separation as the cross‐links. Except for Tx781, the distance distribution of cross‐linked residues significantly differs from the random distribution. B: Fraction of satisfied links with an upper distance bound of 25 Å. C: Fraction of satisfied cross‐links as a function of the upper distance bound. The upper distance bound might be lowered at the expense of cross‐link accuracy.

In the following analysis, we quantify the distance information in the CLMS data. Figure 4(A) shows the distance distribution of cross‐linked residue pairs in the native structure of the cross‐linked CASP targets. With the exception of Tx781, the distance distribution of cross‐linked residues can be clearly distinguished from the distribution of random distances with the same sequence separation and is shifted toward lower distances. Therefore, the cross‐links contain information about residue pairs that are close in space (upper distance bound of 25 Å) which can be used as additional information in protein modeling to restrict the conformational space. Tx781 aggregated during shipping and/or sample preparation which might negatively impacted the cross‐link quality of this target [see α‐carbon distance distribution of Tx781 in Fig. 4(A)]. The fraction of cross‐linked residues below the upper distance threshold of 25 Å is 0.81/0.54/0.75/0.91 for targets Tx767/Tx781/Tx808/Tx812, respectively [Fig. 4(B)]. There are several reasons for long‐distance cross‐links that span larger distance than the specified upper distance bound (25 Å). Conformational flexibility in the protein might lead to cross‐linked residue pairs that are within cross‐link distance in solution, but far apart in the experimental structure. In addition, there are assignment errors from the analysis of mass spectra that leads to wrongly assigned cross‐linked peptides. Another experimental issue is that current data analysis cannot always pinpoint the exact cross‐linked sites at residue resolution. This requires fragmentation evidence for both cross‐linked peptides in the MS2 analysis. However, this fragmentation evidence is not observed for all peptides with the current protocol. If fragmentation evidence is absent, we heuristically estimate the cross‐linked residue pairs using flanking fragmentation events.^17^ Thus, the exact cross‐linked residues cannot be identified in some cases, which results into ambiguous site‐assignments of the cross‐links.

Figure 4(C) shows the fraction of satisfied cross‐links as a function of the accepted upper distance bound. Interestingly, the fraction of satisfied cross‐link distances is still fairly high in the 18–20 Å range. Thus, it might be worthwhile for modeling algorithms to accept a higher fraction of long‐distance CLMS constraints in exchange for more informative cross‐links at a lower estimated upper distance bound. In addition, it would be interesting to further investigate whether few, accurate cross‐links at low FDR or many, less accurate cross‐links at higher FDR are more informative for protein modeling. In another study on human serum albumin, we find that using cross‐links at 10–20% FDR leads to lower RMSD ensembles than 1–5% FDR.^17^ We speculate that structure prediction algorithms that are robust to noise would enable the use of cross‐link restraints at even higher FDR, which would increase the number of available cross‐links from high‐density CLMS experiments even further.

In summary, cross‐links from high‐resolution CLMS contain structural information that is obtainable in approximately 4.2 days of data acquisition for a single protein target. However, we hypothesize that the exploitation of CLMS data by modeling groups could be optimized along three dimensions: 1) Designing error‐tolerant modeling algorithms such that the negative impact of CLMS noise or ambiguous/false assignment is minimized; 2) better estimates or acceptance of higher error at low upper distance bounds of cross‐linked residues; 3) exploiting the geometry of protein models because cross‐links from soluble linking reagents are formed along the surface of the protein structure.^22^, ^23^ Of course, there are likely more ways to exploit the structure information in CLMS data than those we anticipate here.

### Impact of CLMS data on structure prediction in CASP11

**Figure 5:**
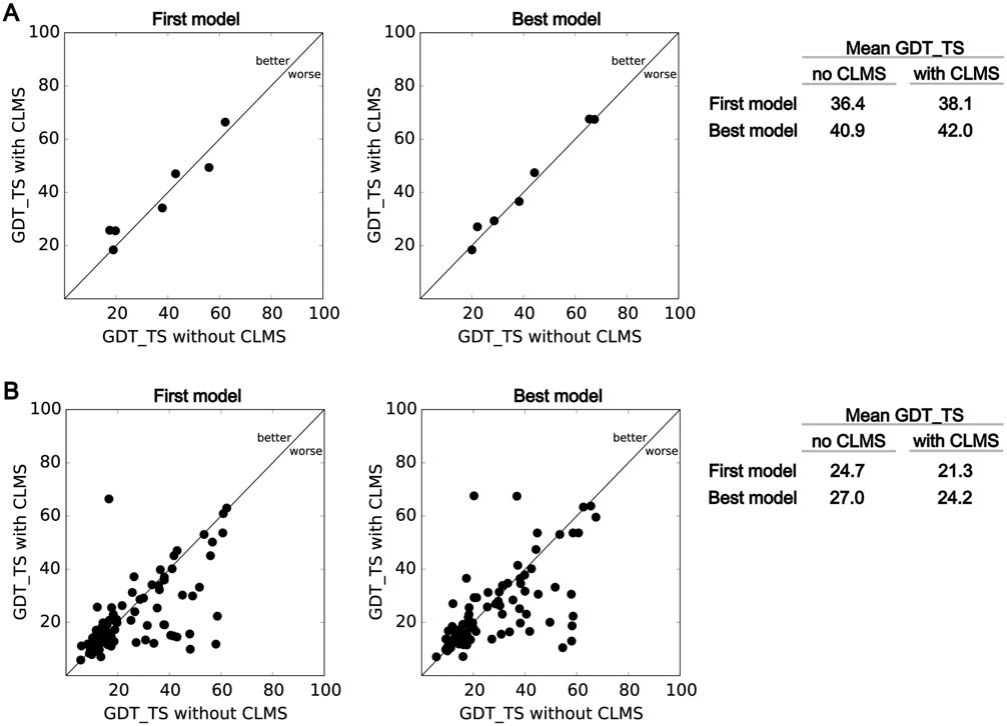
Impact of CLMS data on assisted structure predictions in CASP11. The results of this plot refer to the seven evaluation domains of the four targets Tx767/Tx781/Tx808/Tx812. A: Comparison of the best predicted model of the 19 prediction groups that participated in the regular and assisted experiment. Only groups that participated in both experiments are considered in this plot. Using CLMS data increases the GDT_TS of the best predicted models slightly. B: Comparison of group specific predictions of seven evaluation domains from the 19 prediction group that participated in both experiments. The GDT_TS is lower for assisted models in 53 out of 97 cases, indicating that most prediction groups were yet not able to leverage the structural information in CLMS data. Overall, the CLMS data did not lead to a pronounced improvement of the CLMS‐aided models.

We analyzed the predictions submitted during the CASP11 experiment to measure the impact of CLMS data on model quality. We downloaded the predictions and summary tables from the CASP11 website (http://predictioncenter.org/). We then compared the model quality of predictions submitted to the regular experiment (T0, no CLMS data) and to the CLMS assisted experiment (Tx, with CLMS data). Since we would like to capture the impact of CLMS data, we only compared the best predictions from groups that submitted predictions to both experiments [19 groups, Fig. 5(A)]. Our comparison assumes that these 19 groups used comparable computational methods with and without CLMS data. Therefore, analyzing only the predictions of these 19 groups should give the best estimate of the net effect of CLMS data. In this analysis, the mean GDT_TS of CLMS assisted predictions increases slightly from 36.4 to 38.1 and from 40.9 to 42.0, for first and best‐of‐five models respectively. When removing Tx781 from this analysis, for which the CLMS data acquisition failed (see Figs. 3 and 4), the improvement of the CLMS assisted predictions is slightly higher (mean GDT_TS improvement from 39.7 to 42.8 and 45.6 to 47.8, for the first and best submitted model).

To analyze the group‐specific change in model accuracy, we also analyzed to what extent the groups that participated in both experiments were able to leverage the CLMS data. Figure 5(B) compares the model quality from all predictions groups that participated in both experiments. In 53 out of 97 cases, the predictors submitted models with lower GDT_TS when using CLMS data, the mean GDT_TS drops from 24.7 (no CLMS) to 21.3 (with CLMS) and from 27.0 to 24.2, for the first and best submitted model, respectively. This indicates that most prediction groups were not able to leverage the CLMS data and that the inclusion of this unknown data source rather hurt their modeling approaches. Most likely, the predictors did probably not yet adapt their prediction methods to this new type of data with unknown properties. Two groups submitted superior CLMS supported predictions, which increased the GDT_TS from 36.8 to 67.4 for Tx767‐D1 and 20.2 to 67.6 for Tx808‐D1. The best models of the regular experiment had a GDT_TS of 68.4 and 66.4, respectively. We contacted the groups that submitted the successful Tx predictions and asked whether they exploited cross‐link data in modeling. For Tx767‐D1, the group (McGuffin) employed the cross‐link data in their contact data agreement (CDA) score to select server models for Tx808‐D1, the group (BAKER) reported to us that the critical aspect for the Tx808 target was correct domain parsing, which was not correct in the T0 prediction. Manual inspection lead to a refined domain assignment and domain 1 of Tx808 was then predicted by homology modeling. The CLMS data was not necessary to parse the domains after manual inspection, but confirmed the parsing and individual modeling of Tx808‐D1. These results do not provide clear evidence that CLMS data was helpful for these prediction groups in CLMS‐aided modeling in CASP11 but still point out that CLMS data can be potentially used to assist domain parsing or to select structural models, which is also shown in several earlier studies.^11^, ^12^, ^17^, ^22^, ^24^

It should also be mentioned that CLMS data—at least in this very first inclusion in CASP—did not yet provide a significant advance for the field as a whole. The best models chosen from **all** CASP participants were still better than the best models from the 19 groups using CLMS data (mean GDT_TS from all groups without CLMS data is 40.0 and the mean GDT_TS from the 19 groups using CLMS data is 37.5, for the first model, respectively). However, this is most likely due to the fact that many more groups submitted predictions to the regular experiment (143 groups) than to the CLMS assisted experiment (19 groups). As a result, the chances of finding a higher GDT_TS prediction in the regular experiment was much higher than in the CLMS experiment. In addition, we would like to point out that all measures of model quality lack precision in the analyzed model quality range and that the sample size in this experiment is too small to draw strong conclusions about the effect of CLMS data on prediction quality in CASP11. However, our earlier study showed that high‐density CLMS data enables the reconstruction of the domain structures of human serum albumin, albeit by using CLMS data from 2.1‐2.6 times more acquisitions.^17^ This suggests that improved CLMS data quality could impact CASP predictions in the future.

We believe that this initial experiment demonstrated that CLMS‐driven hybrid methods can be tested in the CASP context. As the experimental protocols are refined and predictors start developing tailored prediction approaches, hybrid methods may provide significant improvements over purely computational approaches in future rounds of CASP.

### Challenges in the CASP11 experiment

The CASP11 CLMS experiment was a success in the sense that it performed a first, truly blind test of a hybrid method for protein structure prediction in a very short timeframe, demonstrating that experimental data acquired in a short time can be used in protein structure prediction, even if the predictions itself were not improved in CASP11.

There were also some lessons learned during the experiment. They should be mentioned to make the reader aware of the challenges of blind testing of hybrid methods as well as to enable improvements for future iterations of CASP.

#### Logistics: planning, communication and shipment/protein aggregation

The logistical challenges of an experimental‐computational experiment are much higher than for a pure computational, because it involves the treatment of physical protein sample. First, the actual proposal for CLMS in CASP was made quite late, which meant that the organization of the experiment had to be made on the fly and whilst the prediction season was already underway. This reduced the time frame for data generation for the experimental group. Since the detection of cross‐link peptides is stochastic and can be improved with additional acquisitions,^17^ we think that an increased data acquisition time would increase cross‐link quantity.

The Rappsilber group also faced the problem of protein sample deterioration. In the case of Tx781 it is likely that the protein sample deteriorated during transit whilst being held in UK customs due to a VAT exemption query.

#### Time constraint

Approximately 4.2 days of data acquisition was performed for each CASP11 target. In addition, the prediction groups only had short time windows for prediction with CLMS data (12–15 days).

#### Novel experimental data with unknown properties

The photo‐CLMS approach generates a novel type of experimental data with properties that are mostly unknown to CASP participants. The coverage, distribution, sparseness, and resolution of the CLMS data are important properties that can be used to develop effective algorithms for protein structure modeling with this type of data. Thus, we speculate that most prediction groups would have been more successful when they would have known the nature of the CLMS data beforehand.

### The future of hybrid method blind testing in CASP

In this section, we would like to suggest some measures that would maximize the impact of the hybrid method blind testing in the CASP setting.

#### Target selection

The careful selection of protein targets will be an important factor in future experiments. We believe that targets should be selected with two goals in mind: 1) Testing the ability of computational methods to model structure with CLMS data for proteins that are well suited for CLMS experiments, and 2) testing a broad variety of different folds to explore biases and issues of CLMS data with certain fold types.

From our experience, we think that α‐helical proteins with ∼200 residues or more seem to be the most suitable targets for current CLMS experiments. The α‐helical structure does not seem to bias cross‐link formation. In addition, α‐helical usually have a larger diameter than β‐sheet proteins, which makes CLMS constraints more informative.

In addition, we recommend the following steps for target selection:

1. Proteins should have sufficient lysines (primary target of CLMS reagents).
2. Proteins should have well distributed digestion sites to ensure uniform coverage with cross‐links, and that digested peptides can be detected by the mass spectrometer because they are not too big. Note that these are current technical limitations that are already actively worked on, and might be overcome eventually. However, for now it would be necessary to actively select targets that are amendable to current CLMS technology.
3. Targets should be from the free‐modeling or hard template based modeling category. This would test whether CLMS data is also useful to disambiguate templates (as shown in prior work by Young *et al*.^12^), or whether the primary application is *ab initio* structure prediction.

#### Computational exploitation of CLMS data

The computational exploitation of CLMS data could be improved in (at least) two ways: 1) Using CLMS data to extract better information from databases, and 2) Leveraging CLMS data in conformational sampling.

Even if the spatial resolution of CLMS data continues to be too low to restrict the conformational space effectively, the data will contain at least some information about the topology of the protein, because adjacent secondary structures (at least α‐helices) should have cross‐links between them. This information could be helpful to select fragments with backbone conformations closer to native. Another possibility for CLMS data is to assist template selection and template alignment of hard template‐based targets.^11^, ^12^ Furthermore, some recent contact prediction algorithms incorporate prior probabilities to improve prediction accuracy.^25^, ^26^ The CLMS data itself or the inferred topology could be used as a prior for contact prediction, which would improve contact prediction accuracy.

Conformational sampling with CLMS data is difficult because of the low spatial resolution of CLMS constraints, which might not sufficiently limit the conformational space. However, tailoring algorithms to the nature of cross‐linking data would compensate this issue to some degree. Cross‐links from soluble linkers are formed along the protein surface and therefore scoring functions should take the surface of the protein into account when modeling CLMS constraints. Xwalk uses breath first search on a surface grid to determine the shortest path between two surface points.^22^ This approach is shown to be more discriminative than constraints that use Euclidian distance, but is computationally expensive and therefore only used as a post‐processing step.^24^ Another study approximates the protein structure by a sphere and measures the cross‐link distance along the arc of the sphere between where the take‐off and landing points are the cross‐linked residues.^23^ This approach loses some resolution of the surface geometry, but is much faster and can be used in conformational sampling. We suspect that other approximations of the protein surface could increase the information content of CLMS constraints while being conformational tractable. Perhaps some answers can be found be by applying algorithms from computer graphics which deals with efficient geometrical representations. We also speculate that one could analyze the residue‐residue distance distribution in many structure decoys to estimate tighter bounds of CLMS constraints with expectation‐maximization type algorithms, such as a Gaussian mixture model.

A different route to increase information content would be to use a lower Euclidian distance bound in modeling. This would increase the number of long‐distance links, but the links that satisfy the distance bound will be more informative. Developing algorithms that are robust to noise will not only be helpful for CLMS data, but also for structure modeling with residue‐residue contacts.

Furthermore, different length of involved side‐chains could be exploited to develop residue specific Euclidian upper distance bounds. However, photo‐cross‐link reagents are highly promiscuous and the fragmentation of peptides is sometimes incomplete. This introduces ambiguity into the site assignment of cross‐linked residues, which needs to be taken into account to develop such residue specific CLMS constraint functions.

Lastly, protein structure prediction algorithms need to carefully weight CLMS constraints with other information from templates, fragments, the energy functions, contacts, and maybe other data from experimental methods.

#### Participation of experimental groups

Additionally, we think that the CLMS‐CASP experiment would benefit from the participation of additional experimental groups. Further experimental groups could blind test their own methods, or rely on the sulfo‐SDA approach described in this article. The latter requires the dissemination of the experimental protocols and software for cross‐link data analysis. Further issues need to be tackled, such as the establishment of the CLMS protocol in a new laboratory that might require proper calibration of mass spectrometers and/or modifications to the cross‐link search software. Experimental groups need to invest a significant amount of staff time and consumables into the experiment, which leaves the open question of how such experiments will be funded in the future. We suggest that experimental groups should be offered authorship in the resulting CLMS‐CASP papers to compensate them for their investment and enable them to request funding for future rounds. Recruitment of more experimental groups is probably the most challenging task toward an improved CLMS‐CASP experiment. However, the experiment would benefit from more experimental groups by a higher number of targets that can be processed. Additionally, the CLMS community would have an opportunity to blind test their tools, which have a wide variety among different CLMS groups. Thus, the independent assessment of CLMS pipelines would have high value to advance this field of study. Finally, it would be highly beneficial to recruit groups that can deliver other types of experimental data for the CASP experiment.

#### Proposal for an alternative testing of hybrid methods

The CASP format introduced rigorous standards into the field of protein structure prediction and can possibly introduce such standards for hybrid methods. The blind testing of computational and experimental methods is important to assess the state of the art of hybrid methods.

However, the most important feature of hybrid methods cannot be tested in CASP in the current setup: The determination of protein structures which cannot be crystallized and solved by NMR. Obviously, because these targets are elusive to traditional structure determination, there would be no structure to evaluate the submitted models in CASP. Thus, using structural models to assess the function of a protein or answer scientific questions would increase the value of hybrid model testing in CASP.

We envision a hybrid method format that does not aim to evaluate the models against experimental structures, which obviously have been amendable to traditional methods. Instead, the goal would be leverage the expertise of the CASP community to convert experimental data to the best structural models available, which are then made public for life‐scientists. Life scientist can verify these structural models by experiment, which would deepen our understanding of these protein systems. Note that our proposal is in line with the direction that CASP assessments are taking. For example, the CASP11 assessors included an assessment of function based on free‐modeling predictions into the experiment; this was successful in two cases.^27^ We are currently discussing specific implementations of this kind of hybrid method testing with the CASP organizers.

We now sketch the setup of this altered experiment. This experiment requires the identification of protein systems for which no determined structures exist and for which models would be most useful to the life science community. This could be accomplished by a specialized board of scientific advisors or an open call in which proposals are reviewed and evaluated. Many experimental groups should be able to provide protein samples, which can then be distributed to groups that are able to provide experimental data. Ideally, this experimental data would be diverse, such as EM, EPR, NMR, and CLMS. Then, the modeling experts of the CASP community would be able to submit structural models. Assessors could use their proven expertise to select the most promising models, which are then published on a web site for life scientists, together with statistics such as the local modeling error. This would generate truly new structural information, leveraging the expertise of experimental groups and the CASP community. Life scientists can these structures to plan mutagenesis experiments or speculate about the molecular mechanisms of this protein.

The modeling community must address several challenges toward this kind of experiment. First, policies for structure prediction depositories must be developed and implemented. The inclusion of experimental data poses additional challenges, such as depositing policies for diverse experimental data. The wwPDB hybrid method task force recently worked out some recommendations for hybrid method repositories and it would be interesting to explore to what degree these recommendations are in line with the planned hybrid method efforts in CASP.^14^ Second, there are challenges pertaining to the identification and acquisition of protein targets and samples as well as the dissemination of protein sample to the experimental groups. Third, a predictive model accuracy evaluation process needs to be developed.

## CONCLUSION

We presented the results of the first cross‐link assisted structure prediction experiment in CASP11. This is the first time in the 22 years of CASP history that the CASP experiment is assisted with actual, experimental data. The experiment was blind to the experimental group and to the prediction groups. For three out of four targets, experimental CLMS data could be acquired that contained accurate structural information in the form of distance constraints between residue pairs with an upper bound of 25 Å. Overall, the CLMS data did not lead to a pronounced improvement in backbone quality of CLMS‐guided predictions.

An experiment that involves the acquisition and release of experimental data faces new issues that need to be addressed by future CASP rounds. We made recommendations for improved CLMS‐CASP experiments that could lead to a larger impact of CLMS data in future CASPs. The rigorous execution and assessment of experimentally assisted predictions in CASP could be of high value to advance the field of hybrid methods.
